# Unraveling Abusive Supervision Climate in Aircrew Workplaces: The Roles of Temporary Organizational Features, Trust, and Gender Dynamics

**DOI:** 10.3390/bs13080656

**Published:** 2023-08-04

**Authors:** Zichan Qin, Timothy J. Lee

**Affiliations:** 1 Faculty of Hospitality and Tourism Management, Macau University of Science and Technology (MUST), Macau 999078, China; 2 College of Hotel and Tourism Management, Kyung Hee University, Seoul 02447, Republic of Korea

**Keywords:** temporary organizational characteristics, abusive supervision climate, trust in supervisor, leader–member exchanges, aircrew

## Abstract

This study aims to advance the theoretical understanding of the contextual antecedents underlying abusive supervision. It provides a fresh perspective on how subordinates’ perceptions of an abusive supervision climate are shaped in temporary work environments. By developing a robust model, this research examines the relationships between temporary organizational characteristics (i.e., temporariness and membership flexibility), trust in ad-hoc supervisors, and perceived abusive supervision climates. We tested the hypothesized model using data from 340 aircrew engaged in temporary and constantly shifting supervisor–subordinate relationships. The results revealed that: (a) temporariness has a direct negative relationship with trust in ad-hoc supervisors, whereas membership flexibility positively affects this trust; (b) the link between temporariness/membership flexibility and a perceived abusive supervision climate is mediated by trust in ad-hoc supervisors. Furthermore, this study highlights gender interactions in a transactional context, indicating that: (c) females perceive a stronger negative association between trust and abusive supervision climates compared to males; and (d) the relationship between temporariness/membership flexibility and trust in ad-hoc supervisors is significant for women and men, respectively. In conclusion, this study underscores the importance of considering the unique organizational characteristics of temporary work settings when examining abusive supervision. It also emphasizes the role of gender in shaping subordinates’ perceptions of a workplace victimization climate, particularly in situations where leader–member exchanges are ephemeral and contractual.

## 1. Introduction

Study of the negative leadership style of abusive supervision has been prominent since the 2000s, with more recent research increasingly focused on this phenomenon [1,2]. The term abusive supervision was coined by Tepper [2] and refers to subordinates’ perceptions of supervisor hostility manifested through sustained verbal and nonverbal mistreatment, excluding physical abuse. Specific instances of these behaviors include yelling, ridiculing, credit stealing, the silent treatment, social ostracism, or venting anger at the individual for others’ mistakes [2,3]. Researchers have documented a number of the deleterious outcomes of abusive supervision on individual mental health, including emotional exhaustion, psychological stress, depression, and others [4]. Similar to the butterfly effect, supervisory abuse can result in substantial economic losses for organizational operations. Factors such as reduced productivity, health care costs, absenteeism, and resignations account for these losses [2,5]. It is estimated that the annual cost of medical treatment for employee depression due to supervisory abuse in US corporations has reached $50 billion [6]. Hence, abusive supervision has become a sensitive but essential issue to investigate within the domain of organizational psychology and the management literature.

Research into supervisory abuse has mostly concentrated on the process of conflict escalation and de-escalation contingent on the long-term social interactions between a fixed supervisor and subordinate [7]. Leader–member exchange (LMX) theory is often used to explain abusive supervision occurrences. The theory postulates that a supervisor influences the paired subordinate through “role-taking, role-making and role-routinization” [8] (p. 248) processes as a sequence of interactions between the two individuals progresses [9]. This understanding assumes that abusive supervision is the outcome of low relationship quality precipitated by sustained negative exchanges between a static hierarchical dyad.

However, this assumption may ignore the phenomenon that organizations are becoming more ambidextrous and flexible to stay competitive in the face of globalization, where boundaries of authority are blurred. One subordinate may report to numerous superiors concurrently [10]. Temporary professional teams (TPTs) such as aircrew cohorts, auditing groups, or film-making projects are often characterized by a limited duration of team formation, a time-bound work mission, and members with diverse backgrounds or expertise in differing disciplinary areas [11]. Individuals in TPTs are often intended to be replaced on a regular basis [10]. Thus, employees in these organizations may not have enough time to go through the three phases of the role-making process that LMX theory contends. The transient leader–member interactions may be terminated at the beginning of the role-taking stage. These circumstances present a new challenge to the classic definition of abusive supervision that emphasizes the sustainability of a supervisor’s animosity. Many supervisory abuse theories and approaches developed in the context of fairly stable and permanent organizational environments may not be applicable to temporary work settings.

More importantly, specific temporary organizational characteristics of TPTs can amplify abusive supervision. Time constraints in team formation reduce the costs for perpetrators, as victims lack opportunities to counterattack [12]. Anticipation of dispersion after task completion also hinders cohesion building, diminishes motivation to communicate, disrupts coordination, and significantly increases interpersonal conflicts [11]. Gale et al. [13] found in a study of 4459 U.S. and Canadian workers that 63% of the cabin crew experienced verbal abuse, with repeated instances being reported. Yang et al. [14] also observed a strong connection between leaders’ abusive behavior and reduced safety performance among pilots in Chinese civil airlines. Therefore, disregarding the likelihood and destructiveness of abusive supervision in temporary professional teams can be risky. Instead, abusive supervision in these ad-hoc working contexts could be perceived as a recurring pattern of hierarchical abuse in multiple supervisor–subordinate relationships wherein subordinates’ perceptions of abuse rely on a sequence of linked experiences [15]. In other words, abusive supervision in TPTs is sustained from an organizational perspective.

To achieve a more comprehensive understanding of abusive supervision across diverse organizational structures, this study focuses on the contextual dynamics underlying abusive supervision in temporary professional teams. The study specifically investigates aircrew workplaces where airline roster systems allocate crew compositions randomly based on their rest time and qualifications, resulting in transient and variable team compositions [16]. Firstly, it examines the relationships between temporary organization characteristics (i.e., temporariness and membership flexibility) of TPTs and subordinates’ perceptions of an abusive supervision climate. The focus lies in how abusive supervision perceptions emerge in transient and fluid supervisor–subordinate relationships, as opposed to a static relational dyad. Secondly, the study proposes that these contextual features of TPTs could be linked to a perceived abusive supervision climate via the mediating effects of trust in ad-hoc supervisors. Lastly, the role of subordinate gender in supervisory trust-building and perceptions of an abusive climate precipitating are also investigated. This recognizes the significance of gender cues as prominent indicators for subordinates when deciding whether to trust an unfamiliar supervisor in a short-term relationship. Therefore, this study aims to provide new insights into the context-specific antecedents of supervisory abuse in temporary work settings by examining the underlying relationships between temporary organizational characteristics, gender differences, and the perceptions of abusive climates. These understandings may serve as a basis for future empirical research in similar contexts where abusive supervision accumulates from dynamic leader–member exchanges. In practice, it will provide a lens through which project practitioners or ad-hoc group leaders can formulate feasible and effective management strategies to address the issue of supervisory abuse in temporary working environments. These strategies will be particularly salient for project settings, such as consulting groups, medical emergency action teams, or other provisional sectors, thereby promoting project success.

## 2. Literature Review

### 2.1. Perceived Abusive Supervision Climate in Temporary Work Settings

A definition of abusive supervision has been extensively cited as “subordinates’ perceptions on the extent to which their supervisors engage in the sustained display of hostile verbal and non-verbal behaviors, excluding physical contact” [2] (p. 178). An emphasis on the feature of abusive supervision is a “sustained display of non-physical hostility” [17] (p. 265). This implies that subordinates are continuously exposed to a specific supervisor’s malice through a history of hierarchical mistreatment. Tepper (2007) gave an example of “a boss who has a bad day and takes it out on his or her subordinates by exploding at them would not be considered an abusive supervisor unless such behavior became a regular feature of his or her repertoire” [17] (p. 265).

In such a case, the one-off interpersonal mistreatment of a subordinate by a supervisor has been excluded from the notion of abusive supervision. The frequency and longevity of supervisory abuse has also been measured on the basis of long-term interactions between a subordinate and the immediate supervisor [18,19]. Six months is the most widely used length of time for measuring same-leader abusive behavior [3,20]. A one-year period has also been applied as a minimum requirement for the collection of employee supervisory abuse intensity ratings in a fixed hierarchical dyad [21].

However, a criterion that stands on an assumption of sustained supervisory mistreatment neglects the likelihood of abusive behaviors in temporary work settings. This is especially so where supervisor–subordinate connections are transitory and continuously shifting, such as aircrew workplaces, construction groups, and software engineering teams [10]. In these contexts, the development of leader–member exchanges are stagnant. Often subordinates lack formal and reciprocal power (i.e., the relational impacts on others accrued through the social contacts), as LMX remains in the ‘stranger’ phase [22,23]. As a result of the followers’ greater reliance on leaders to achieve projected targets, they may perceive a substantial power imbalance, which prevents them from standing up to the wrongdoers. Empirical studies have confirmed this premise. Ariza-Montes et al. [24] found that temporary hierarchical interrelations and elastic work arrangements were associated with an increased prevalence of bullying. Di Martino [25] mentioned that temporary employees in the healthcare sector are at a higher risk of experiencing stress and workplace violence due to a lack of job autonomy and perceived vulnerability vis-à-vis their superiors.

Prior studies have indicated that employees frequently extrapolate the treatment they receive from their supervisors to the entire organization, as supervisors are representatives of the organization [26]. This phenomenon could be more applicable to TPTs, where supervisors are often replaced on a frequent basis, thus lowering the salience of individual personalities. Employees may attribute the harsh treatment they receive from supervisors to the organization and perceive organizational cruelty. However, viewing abusive supervision as an organizational phenomenon is far more damaging than interpersonal abuse and can manifest as organizational-deviant behaviors such as service sabotage, unsafe behaviors, or employee theft [27,28]. This discussion suggests that further research into the methods and perspectives of how supervisory mistreatment emerges in temporary grouping forms is required. This research may vary from the current understandings of workplace abuse, as they conceptualize bullying as an escalating process of interpersonal conflicts between the relatively permanent dyad (i.e., the focus being on individual dispositional traits rather than organizational phenomena) [7,29].

Understanding non-persistent abusive supervision in temporary working contexts should not concentrate solely on the individual leader’s behavior but also on the interpretations of subordinates exposed to the supervisor(s)’ repeated hostile behaviors. Zhang and Liu (2018) suggested that the continuity of exposure to hostility is ultimately determined “in the eye of the beholder” [30] (p. 725). The same behaviors from an abusive supervision repertoire could be appraised as ‘hostile’ in one current state but interpreted as ‘motivating’ or ‘strict guidance’ in another. This may be the case where a subordinate has experienced a post-traumatic growth [31]. It may also be prominent in TPTs where a subordinate may be subjected to repetitive or similar abusive behaviors from ever-changing supervisors rather than the same individual. Hence, even if the objective existence of supervisory abuse could be independent from subordinates’ perceptions, “the absolute truth is nowhere to be found” [32] (p. 253).

Drawing upon social information processing theory [33], unexpected events trigger a greater sensemaking process than expected ones. When confronted with linked abuse experiences, accumulated through dynamic supervisor–subordinate interactions, a subordinate is more likely to engage in sensemaking processes, resulting in shared and communal ideas of abusive supervision at the climate level. Hence, the construct of “perceived abusive supervision climate” (PASC) [33] (p. 1513) encompasses employees’ integrated perceptions of abusive behaviors within their work unit. It is created to shape reality so as to make it predictable, approachable, and explicable [34]. This concept of PASC allows for the quantification of subordinates’ ratings of intermittent supervisory abuse exposure in temporary work settings.

### 2.2. The Contextual Antecedents Underlying Perceived Abusive Supervision Climates

#### 2.2.1. The Rationale for Temporary Professional Teams

Temporary professional groups or teams (TPTs) are defined as an aggregation of members whose attitudes, capabilities, and skills enable teams to temporarily cooperate to complete shared tough or stressful goals [35,36]. The transitory structure of teams is commonly seen in projects, ad-hoc groups, or special operations teams, such as cockpit crews on flights, cross-functional management groups in the hotel sector, or emergency action teams in a medical trauma center [37,38]. The literature highlights six characteristics of temporary professional teams (TPTs) that differentiate them from permanent organizational structures:

- TPTs are grounded by “an ex ante limited duration” [39] (p. 26) and all the members are aware of the time of dispersion;
- Standardized roles and well-trained individuals mean that group members change frequently [10,38];
- Members have little experience working together and low expectations of future encounters [35];
- Members with cross-functional, diverse backgrounds or skills are interdependent on each other’s experience or expertise to achieve the mission [10,38];
- Tasks are often non-routine and involve high stakes or stressful goals [10]; and
- Ambiguous hierarchies are often configured in TPTs with members who have both intrinsic roles within the temporary group and extrinsic roles outside the group, which may lead to an “authority gap” [10] (p. 3). Team leaders cannot decide promotions as superiors usually do in permanent structures.

TPTs range from ‘general’ to ‘pure’, having one or more of the six features. However, the categorization of aircrew workplaces are not captured by descriptors such as ‘non-routine tasks’ and ‘ambiguous hierarchies’ due to well-defined and stable aircrew assignments, working procedures, and promotion mechanisms. Nevertheless, all types of TPTs share the common feature of having a finite duration for team combinations [39]. This study focuses on two organizational characteristics, namely ‘temporariness’ and ‘membership flexibility’. These characteristics allow an investigation of abusive supervision climates within TPTs, as these features result in transitory and constantly shifting team compositions. Temporariness refers to a limited duration of group configuration or predefined dispersal upon mission completion [39]. Membership flexibility relates to the interchangeability or replaceability of personnel with similar expertise or experience [40]. This interchangeability occurs primarily in high-performance teams with a great extent of role clarity and comprehensive organizational training.

#### 2.2.2. The Trust-Mediated Links between Temporariness and PASC

Trust in supervisors indicates the extent of “subordinates’ willingness to undertake risks based on positive hope towards their supervisors’ intentions” [41] (p. 135). It is composed of two levels, namely cognitive trust and affective trust. The cognitive dimension of trust refers to the subordinates’ consequent willingness to take risks as a result of good impressions of superiors’ job-related performance [42]. It is transactional by nature and cannot be established unless the subordinate perceives the superior as possessing reliable knowledge and aptitudes to perform supervisory duties [41]. However, the temporariness of TPTs make supervisors aware of the impending termination of the tasks, resulting in a focus on the immediate deliverables required before the predefined time of dispersion [36]. Such task-oriented cognition may impede efforts to establish trust and positive relationships with subordinates. Brief, discontinuous, and alien ties formed in TPTs might be seen as less valuable and trivial [43]. An authoritarian leadership style may be deemed to be useful to ensure subordinates are more compliant and efficient in quickly ‘getting the job done’ [44]. Such conduct may further lower subordinates’ favorable perceptions of supervisors’ job-related performance, enhancing a sense of leaders’ incapacity. These perceptions may hinder subordinates from developing cognitive trust with the ad-hoc superiors, which may result in more reports of abusive supervision.

Another affective dimension of trust represents followers’ benign hopefulness towards leaders’ motivations and their readiness to tolerate the leaders’ vulnerabilities. This tolerance is the result of emotional bonds and social intimacy developed over time between the two parties [42]. In other words, subordinates with a strong affective trust in supervisors would believe that the supervisors would never sacrifice others for their own personal interests. This belief stems from a long-term positive reciprocity [9]. Thereby, when individuals experience criticism or negative comments, they are more likely to be introspective rather than blame the leader [41]. This suggests the functioning of affective trust in supervisors may extend subordinates’ positive interpretations of supervisory behavior, even if the behavior is part of an abusive supervision repertoire.

The short-term grouping process of TPTs provides less time to learn about individual expertise or interests, resulting in transactional exchanges and difficulties in forging affective ties between supervisors and subordinates. In addition, a recognition of dispersion time may reduce supervisors’ expectations of future interactions with subordinates. This may result in reduced support at work and lead to a lessening of affective trust. As a cabin crew stated, “I am always flying with new colleagues...You are with this group of people but, you know, essentially you are on your own as well...” [45] (p. 220). In such instances, if the supervisor engages in offensive behaviors, subordinates may believe they are the victims of a conflict of interest and that the leader’s intention is ‘deliberate’ or ‘hostile’ to maximize their own benefits. Based on this discussion, the following hypotheses are constructed:

**Hypothesis H1.**  *Temporariness has a negative effect on the subordinates’ trust in ad-hoc supervisors within TPTs.*

**Hypothesis H2.**  *Subordinates’ trust in ad-hoc supervisors mediates the positive relationship between temporariness and PASC within TPTs.*

#### 2.2.3. The Trust-Mediated Relationships between Membership Flexibility and PASC

A distinguishing feature of TPTs is membership flexibility. This indicates that members of TPTs are interchangeable based on organizational role clarity. Anyone with the same qualifications can perform the other’s jobs; thus, team members are intended to be replaced on a regular basis [10]. TPTs’ institutionalized socialization tactics frequently standardize role expectations and job demands for new recruits in order to be more efficient and effective in dealing with unpredictable and time-bound duties. Through sequential and fixed practices, all recruits are informed of the sequence of actions leading to task mastery, and thus experience less anxiety and uncertainty about task completion [46]. For example, crew resource management training programs are often used to exercise inter-group teamwork and communication in the aviation industry [47]. Prior to formal flights, pilots are required to perform emergency procedures on a simulator with other copilots and commanders. Flight attendants also practice in-flight service and evacuation procedures in groups. As a result, members are aware of both their own and coworkers’ role expectations, allowing interchangeability if workers possess the same qualifications or expertise.

TPT membership flexibility built upon high role clarification enables members to have clear conceptions of each other’s job demands and responsibilities. The understanding that coworkers can demonstrate task performance competence increases confidence in the team. As such, it addresses the practical components of work and the functional (as opposed to socioemotional) aspects of interpersonal relationships within the work unit. Despite the absence of traditional trust foundations such as familiarity, shared experience, and sustained exchanges in this temporary working system [35], followers’ role-based trust in superiors may emerge swiftly, as they first rely on the leader as a supervisory role rather than as a distinct individual.

Meyerson et al. [38] claimed swift trust emerges when implicit threats and role clarity coexist within temporary systems. That is, an individual would be willing to expose vulnerabilities and invest trust in one another in a short period rather than waiting for it to grow over time. This trust is based on a clear understanding of the other party’s roles and the aim of cooperation to deal with pressure or high risks. This phenomenon has been termed the “initial trust paradox” [48] (p. 474); more specifically, it suggests that when circumstances involve implicit threats, subordinates are willing to trust supervisors in the new and short-lived relationship, since role expectations are key clues that they can rely on [41], as Meyerson et al. (1996) highlighted:

*We trust engineers because we trust engineering and believe that engineers are trained to apply valid principles of engineering; moreover, we have evidence every day that these principles are valid when we observe airplanes flying. We trust doctors because we trust modern medicine, and we have evidence that it works when antibiotics and operations cure people* [38] (p. 173).

In light of this, TPT membership flexibility based on high role clarity boosts subordinates’ confidence in supervisor competency, proficiency, and dexterity, and thus fosters cognition-based trust in ad-hoc supervisors. To achieve TPT tasks involving implicit threats, subordinates with swift trust are more prone to believe supervisors’ positive intentions, shifting criticism from personal to mission-related issues. Thereby, they perceive less abusive supervision. Based on this discussion, the following hypotheses are posited:

**Hypothesis H3.**  *Membership flexibility has a positive effect on subordinates’ trust in ad-hoc supervisors within TPTs.*

**Hypothesis H4.**  *Subordinates’ trust in ad-hoc supervisors mediates the negative relationship between membership flexibility and PASC within TPTs.*

### 2.3. Probing into the Moderating Effects of Subordinate Gender

The gender of the interacting dyad becomes a fundamental and salient cue in interpersonal relationships. It has a significant influence on social information processing towards their own and others’ behaviors, especially when the two parties are strangers in temporary working contexts [49]. Research has also highlighted that males and females have different attitudes and appraisals of affective events that occur in the workplace. The development of employees’ trust in leaders and perceptions of an abusive supervision climate may vary based on their gender perspectives [50]. Hence, an examination into the gender effects underlying abusive supervision dynamics is also warranted.

McAllister [51] argues that cognitive trust is rational in nature. It refers to an objective assessment of the other party’s functional characteristics, such as competence, honesty, integrity, quality, and reliability [51]. However, the nature of affect-based trust is relational. It refers to the emotional bond that exists between individuals and is based on reciprocal concern, compassion, and consideration. It underscores empathy, belongingness, and rapport, and forms a mutual respect for the other party. Contingent on social role theory, soft, nurturing, sensitive, and relationship-oriented traits are usually ascribed as being feminine [52,53]. Females tend to perceive interactions communally and have expectations of forming long-term social bonds, making them more altruistic but also vulnerable over the short term [54]. Female subordinates may value affective trust more than cognitive trust when compared to male followers. However, the transient nature of TPT configurations precludes the provision of relational cues, such as prior familiarity, open communication, perceived consideration, and assisting behaviors, necessary for members to build affective trust in supervisors [46]. A recognition of impending termination also indicates that the current connection with the supervisor may not develop or last. As a result, women who place a higher value on affect-based trust may be more cautious and less willing to trust than men in a short-lived relationship. In these circumstances, females are more likely to experience mistrust or even negative attitudes towards the source of strangeness [49].

Masculinity is stereotypically associated with competitive, task-oriented, and agentic (i.e., result-based) traits [53]. Males make more instrumental decisions about trust and view interactions more functionally. They have higher expectations of return on transactional exchanges, and thus would be more willing to take higher risks. They are more likely to trust strangers as long as they found cues denoting the trustees were willing to be involved in trustworthy actions, implying a greater likelihood of returning profitable outcomes [54]. Male subordinates are prone to value cognition-based trust more than affect-based trust when compared to females, because cognitive trust is associated more with result-oriented attributes. Given that TPT membership flexibility is built upon role clarity and results in a clear understanding of each other’s job proficiency and task mastery [46], cognitive trust related to supervisors’ dependability is increased. Hence, male trust is predicted to be more prevalent in flexible, rational, and role-based circumstances, whereas female trust is more likely to develop in long-term relationships following the formation of a close or intimate social bond. Based on this, the following hypotheses are established:

**Hypothesis H5.**  *Subordinate gender moderates the connection between temporariness and trust in ad-hoc supervisors, such that the negative relationship between temporariness and trust in supervisors is stronger among female than male subordinates within TPTs.*

**Hypothesis H6.**  *Subordinate gender moderates the association between membership flexibility and trust in ad-hoc supervisors, such that the positive relationship between membership flexibility and trust in supervisors is stronger for male than female followers within TPTs.*

Consistent with this discussion, women are stereotyped as more communal or relational in their ‘self-construal’ [53]. They place a higher priority on social harmony and make a greater effort to maintain relationships than men do. Women are also said to be more likely to weep and report feeling angry, frustrated, fearful, anxious, or underappreciated (hurt and rejected) than men [50]. Female employees may therefore be more sensitive to affective events occurring at work and more prone to associate the sentiments or emotions induced by such incidents with their perceptions of the overall work unit climate. When experiencing a superior’s offensive behavior, a female with less trust in the supervisor may take the behavior seriously and view it as a psychological contract breach within a social exchange, and thus react to it more intensively [55]. On the contrary, males tend to distinguish themselves by making obvious their autonomy, independence, and personal achievements [56]. Male employees are more inclined to place a higher value on tangible rewards in the workplace, such as remuneration or promotion, while social rewards like integration, reciprocal trust, and close relationships are considered less salient. Therefore, even if male followers lack trust in supervisors, they will be more task-oriented and concentrated on self-determination. They will also place less value on the quality of exchanges or social support. Males may view supervisory verbal assaults as less detrimental to their actual interests. As a result, this study proposes that the effect of trust in ad-hoc supervisors on abusive supervision climate perceptions is likely to be stronger for female subordinates than for their male counterparts.

**Hypothesis H7.**  *The negative relationship between trust in ad-hoc supervisors and PASC is stronger for females as opposed to males.*

**Figure 1:**
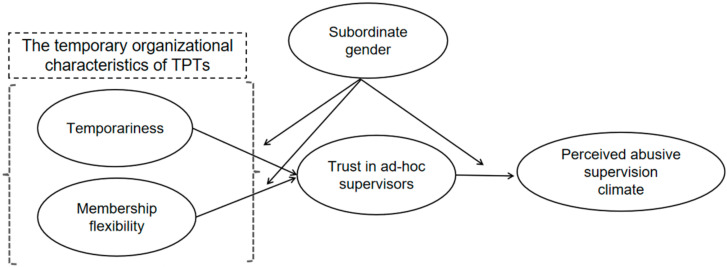
Hypothesized theoretical model.

Based on the discussions, Figure 1 presents the hypothesized theoretical model.

## 3. Methodology

### 3.1. Research Design and Sampling

The study employs a quantitative approach, as it is useful for generating “meaning through objectivity uncovered in the collected data” [57] (p. 66). It also facilitates an understanding of the underlying reasons behind a phenomenon within a particular group [58]. We collected subordinates’ online self-report ratings of the study variables.

Purposive sampling was administered, as the sample needed to include participants who have relevant and varied viewpoints on the ideas and issues under consideration [59]. Four criteria determined the research sample: First, the target population should be individuals who are involved in dynamic supervisor–subordinate relationships within TPTs, as this study aims to investigate non-persistent abusive supervision in a temporary work setting. Second, having highlighted subordinate dominance in supervisory behavior evaluation, subordinate perspectives are the focus. Third, various organizational sizes ensure sufficient variance of ‘temporariness’ and ‘membership flexibility.’ Last, a subordinate’s sensemaking of an abusive climate is accumulated through interactions with ever-changing supervisors over time. Thus, the final criterion requires at least six months of tenure, during which supervisors may have changed. As a result, this study purposely recruited a non-managerial aircrew workforce, namely flight attendants and pilots involved in transient and flexible supervisor–subordinate relationships, whose tenure was full-time for at least six months in either large airlines or with small regional carriers.

### 3.2. Procedure

As one of the researchers was a former flight attendant, she was able to reach respondents from aircrews. The comradeship allowed her to obtain their trust and voluntary participation in this project. With the assistance of the key informants, the sample data were collected from first-line non-managerial airline personnel in eight airlines from January to July of 2022.

The integrative survey, encompassing all established constructs, was administered through the ‘wenjuan.com’ online survey platform. This platform offers questionnaire creation, release, data collection, and analysis services to enterprises and researchers. The instrument includes both an English version and a back-translated Chinese version. Leveraging a bachelor’s degree in English language and literature, the first author utilized her expertise to adjust the wording and expression in the English version to enhance comprehensibility within the Chinese linguistic context. The researchers formed a discussion group to evaluate the translated elements and made revisions based on predetermined criteria. The draft was then reviewed by a small number of aircrew and a statistics expert to assess the clarity and suitability of item settings for data analysis. Based on this feedback, redundant items were eliminated, and response choices were revised to achieve face and content validity [60]. A revised version of the questionnaire was constructed.

The online link for the questionnaire was then distributed to the potential participant groups of 1031 aircrew. For each completed survey, they were provided with a flying keepsake as an incentive. As a result, we received 351 completed surveys, with a response rate of 34.04%. Eight respondents with less than six months tenure and three participants in other job types were deleted. Ultimately, 340 valid questionnaires were achieved. A total of 162 respondents were male and 178 were female. The majority (75.3%) of them were aged between 26 and 30 years old. The average organizational tenure was 4.15 years (SD = 2.569). There were 192 flight attendants and 148 pilots. The majority of employees held a college diploma or bachelor’s degree. The breakdown of business ownership was 67.9% large state-owned airlines, 25.9% small regional carriers, and 6.2% were joint ventures.

### 3.3. Ethical Considerations

The study was conducted in adherence to the principles outlined in the Declaration of Helsinki, thereby upholding high standards of ethical research. The research protocol underwent rigorous evaluation by the Faculty of Hospitality and Tourism Management at Macau University of Science and Technology. Participants were informed that the data collected were to be used exclusively for academic purposes. To ensure transparency and uphold ethical standards, an informed consent form seeking their agreement to participate in the research was administered to the participants. This consent form explicitly outlined the nature of the tasks involved and assured confidentiality and anonymity.

### 3.4. Measurement

Due to the transient nature of supervisor–subordinate relationships within aircrews, there was no fixed immediate supervisor. Thereby, most statements were prefaced with ‘the purser (or captain)’ instead of the original term ‘immediate supervisor/boss.’ Each scale item was measured using a five-point Likert scale ranging from 1 (totally disagree or false) to 5 (totally agree or true).

#### 3.4.1. Temporariness

Temporariness was measured by the ‘temporary duration item’ from the ‘Temporariness’ scale [39]. The subject, being ‘the project,’ was replaced with ‘my relationship with the purser/captain’ and rephrased according to the nature of the aircrew profession. An exemplified prompt would be “I am aware that once the destination is reached, my relationship with the purser (captain) would be dissolved” or “Already at the beginning of each flight task I knew that my relationship with the purser (captain) would not exist in the long run.” The Cronbach’s alpha value for this scale was 0.77.

#### 3.4.2. Membership Flexibility

To evaluate the flexibility of memberships in TPTs, this study used the ‘member flexibility items’ from the Work Group Characteristics Measure, which was revised to accommodate the aircrew working context [40]. An example could be: “The aircrew compositions are very flexible in terms of changes in membership” or “Members of aircrew teams who have the same qualifications can easily replace/fill in for one another” (Cronbach’s α = 0.67).

#### 3.4.3. Trust in Ad-Hoc Supervisors

Subordinates’ trust in ad-hoc supervisors was measured with the five-item subscales of ‘salesperson trust of sales manager’ from the Reciprocal Trust Measurement [61]. This measurement scale includes both cognitive trust and affective trust and has indicated a high reliability. Instances were “I have complete trust that supervisors who play the purser(captain) role will treat me fairly” and “I feel free to discuss work problems with supervisors who play the purser (captain) role without fear of having it used against me later”. The Cronbach’s α for this measure was 0.89.

#### 3.4.4. Subordinates’ Perceptions of an Abusive Supervision Climate

Subordinates’ ratings of the frequency of a specific supervisor’s non-physical hostile behaviors were not examined in this study. The study instead operationalized abusive supervision climate perceptions as follower evaluations on past exposure to supervisory abuse in dynamic supervisor–subordinate interactions within a temporary work unit. Tepper’s [2] 15-item scale served as the primary reference. Temporary working contexts were incorporated into the original scale by adding the prefix, namely “In the airline where I work”. It also replaced frequency responses with the choices indicating the degree of agreement, ranging from 1 (strongly disagree) to 5 (strongly agree). Examples of questions were: “In the airline where I work pursers/captains were often ridiculing me” and “In the airline where I work pursers (captains) were often putting me down in front of others” (Cronbach’s α = 0.94).

#### 3.4.5. Control Variables

In light of the literature review, age and tenure were kept constant because they had been discovered to affect subordinates’ perceptions of supervisory abusive behavior [62]. Job types were controlled as well, because relationship-oriented occupations were also found to be more associated with abusive supervision in comparison to non-relationship dealing jobs [63]. Age and job types were coded as dummy variables (e.g., pilot = 0 and flight attendant = 1). Tenure was measured by the year of service reported by respondents.

### 3.5. Analyses

Structural equation modeling (SEM) was employed to analyze the data using the Amos 24.0 program. Initially, a confirmatory factor analysis (CFA) was conducted to evaluate the measurement model’s validity and determine the dimensionality and discriminant validity of the scales [64]. A path analysis was then performed to investigate the impacts of the independent variables of temporariness and membership flexibility on perceived abusive supervision climate. Using the bootstrapping method (5000 times) to estimate confidence intervals, the mediating effects of trust in ad-hoc supervisors were examined [65]. This approach avoids assuming that the two regression coefficients’ product follows a normal distribution by drawing a great number of replacement samples from the collected data. In addition, Hayes’ [66] PROCESS macro was used to test the moderated effects of subordinate gender.

## 4. Results

### 4.1. Descriptive Statistics

A correlation analysis between the constructed variables was undertaken and the expected zero-order correlations between seven variables were sufficient (see Table 1). However, we found that the conjectured connection between age and abusive supervision climate perceptions was non-significant.

### 4.2. Measurement Model

To examine the composite reliability and discriminant validity of the latent variables, a CFA was conducted with the method of maximum likelihood estimation [67]. The results indicated that the four-factor model was a good fit with the collected data (χ^2^ = 435.45, df = 221, χ^2^/df = 1.97; *p* = 0.000; GFI = 0.90, TLI = 0.95; CFI = 0.95; RMSEA = 0.05; and SRMR = 0.05). The goodness-of-fit and badness-of-fit indicators conformed to the strict cut-off values established by Hu and Bentler [68]. Factor loadings achieved a statistically significant level (*p* < 0.001) ranging from 0.51 to 0.90. Four alternative models were tested (See Table 2). The findings showed that the proposed model was distinct from the competing models and yielded the best-fit indicators. Discriminant validity was assessed by comparing the square root of AVE for each construct with its correlation with other latent variables [64]. The results suggested that individual constructs provided a good discriminant validity as the values of diagonal elements were more than those of the off-diagonal elements (Table 3). Hence, these results showed that the established measurement model is both reliable and valid.

### 4.3. Hypotheses Testing

Having examined the measurement model, the hypotheses were evaluated using a structural model. As mentioned above, age did not have a significant relationship with perceived abusive supervision climate and was therefore removed as a control variable. However, it did control the effects of tenure and job types on the dependent variable in the structural model. The results suggested that the removal of job types was acceptable, as the path coefficient between job types and abusive supervision climate perceptions was not statistically significant. Job types were thus excluded but tenure was kept as the only control variable. The findings demonstrated that this structural model fit the data well (χ^2^ = 502.11, df = 243, χ^2^/df = 2.07; *p* = 0.000; CFI = 0.94; TLI = 0.94; RMSEA = 0.06; SRMR = 0.06). The results revealed statistically significant path coefficients between temporariness and trust in ad-hoc supervisors (β = −0.31, *p* < 0.001) and between membership flexibility and trust in ad-hoc supervisors (β = 0.28, *p* < 0.001), thereby supporting Hypotheses H1 and H3, respectively.

**Figure 2:**
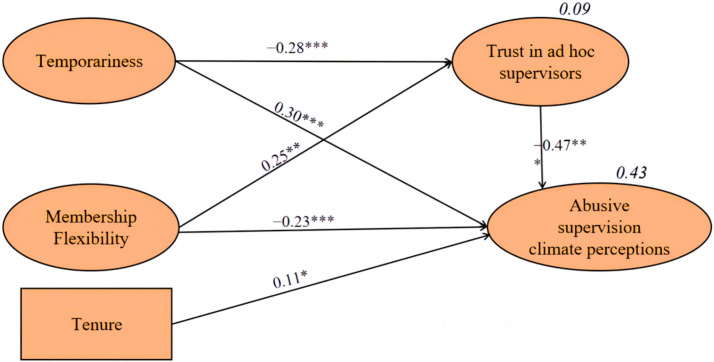
Standardized results of the mediated model. Note. Squared multiple correlations for endogenous variables are reported in bold and italics. *** *p* < 0.001, ** *p* < 0.01, * *p* < 0.05.

To investigate the full, partial, or non-mediating effects of trust in ad-hoc supervisors, temporariness and membership flexibility were then directly linked to PASC [69] (see Figure 2). A bootstrapping procedure (5000 times) was performed to estimate the significance of the indirect effects. The path coefficient between trust in ad-hoc supervisors (TRUSTS) and perceived abusive supervision climate was sufficient (β = −0.47, *p* < 0.001). The mediating effect of TRUSTS on the relationship between temporariness and PASC was also supported (point estimate = 0.09, *p* < 0.001; bootstrap 95% CI [0.04, 0.15], SE = 0.03). Likewise, the connection between membership flexibility and abusive supervision climate perceptions via the mediator of TRUSTS was validated (point estimate = −0.13, *p* < 0.001; bootstrap 95% CI [−0.24, −0.05], SE = 0.05). The structural model explained a substantial 43% of the variance in PASC. Therefore, Hypotheses H2 and H4 were both supported (See Table 4).

### 4.4. Test of the Moderating Role of Subordinate Gender

The study also examined the moderating effects of subordinate gender on the relationships between the two temporary organizing characteristics and trust in ad-hoc supervisors, as well as the effect of TRUSTS on perceived abusive supervision climate. The results of hierarchical regression analyses indicated that the interaction term (i.e., temporariness × subordinate gender) had a significant effect on TRUSTS (β = −0.16; *p* < 0.05). The interactive effect of subordinate gender and membership flexibility on TRUSTS was also statistically significant (β = −0.27; *p* < 0.01). Likewise, the results showed the interactive term (TRUSTS × SG) on PASC was also sufficient (β = −0.32; *p* < 0.01) (See Table 5).

**Figure 3:**
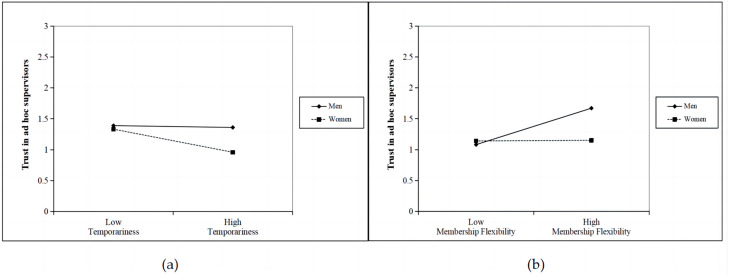
(**a**) Interaction between temporariness and subordinate gender on trust in ad-hoc supervisors; (**b**) Interaction between membership flexibility and subordinate gender on trust in ad-hoc supervisors.

**Figure 4:**
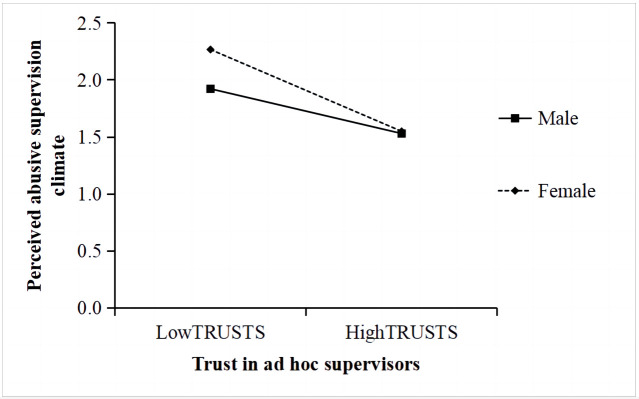
The interaction between trust in ad-hoc supervisors and subordinate gender on PASC.

To understand the directions of significant interactions, we then conducted moderated mediation regression equations separately for female (coded as 1) and male (coded as 0) subordinates. We examined the simple slopes at one standard deviation above and below the mean of the moderator. The results confirmed our predictions. Specifically, the negative relationship between temporariness and TRUSTS was significant only among female followers (t = −4.07, *p* < 0.001). Conversely, the positive association between membership flexibility and TRUSTS was significant only for male subordinates (t = 5.09, *p* < 0.001). Furthermore, the findings indicated that the negative effect of TRUSTS on PASC was stronger for females (t = −9.16, *p* < 0.001) compared to males (t = −3.50, *p* < 0.001). Therefore, these interaction patterns provided support for Hypotheses H5–H7 (See Figure 3 and Figure 4).

## 5. Discussion

### 5.1. Theoretical Implications

This study contributes to abusive supervision research in several significant ways. First, it uncovered novel contextual antecedents underlying abusive supervision dynamics. It provides an explanation for how the temporary organizational characteristics of TPTs (i.e., temporariness and membership flexibility) shape subordinates’ perceptions of an abusive supervision climate. Research has mainly assumed a ‘giver’ and a ‘taker’ paradigm when investigating abusive supervision in relation to the individual predictors of supervisors and subordinates. However, this approach may ignore the implication of context on the supervisor–subordinate interactions as well as the subordinates’ judgement of supervisory behavior. Aquino and Thau (2009) have argued that “our review of studies comparing various forms of victimization as a function of occupation or work sector does not present a clear conclusion about what types of organizations or job sectors are likely to be associated with higher victimization” [70] (p. 726). Thereby, the findings of the present study have advanced the theoretical underpinnings of how the contextual antecedents of temporary organizing features underpin an abusive supervision climate.

Second, this study explores abusive supervision in temporary work environments, shedding light on perspectives that differ from those applied to permanent work settings. Traditional leadership theories attribute abusive supervision to long-term interactions between a paired supervisor–subordinate, emphasizing sustained animosity, continuous relationship dynamics, and escalated leader–member exchanges [8,71]. However, businesses in the 21st century require agility and adaptability to effectively respond to changing circumstances. Temporary organizational structures are increasingly used to establish organizational dexterity where fixed hierarchical dyadic relationships may not endure [10]. Consequently, many theoretical approaches that assume abusive supervision results from sustained hostility by a specific superior cannot be fully applied to these contexts. Research has, however, substantiated the occurrence and detrimental effects of abusive supervision in temporary workplaces [13,14]. The complexity and significance of leadership in temporary organizational structures also warrant addressing abusive supervision in these settings [10]. The current study initiated an investigation of non-persistent abusive supervision in temporary work settings. It extends the context-free concept of abusive supervision from individuals’ evaluations of their immediate supervisor’s abusive behaviors to a more dynamic and context-specific notion of ‘perceived abusive supervision climate’. This perception is contingent on a member’s evaluation of past exposure to intermittent abusive supervision from ever-changing supervisors.

This study further addressed how temporary organizational characteristics of TPTs relate to perceived abusive supervision climates by suggesting the mediating effects of trust in ad-hoc supervisors underlying relationships. The findings revealed that temporary grouping processes can lead to members having less trust in ad-hoc supervisors due to a lack of time to learn about each other’s expertise and develop social bonds. Meanwhile, the impending dispersal of the group may sap supervisors’ motivation to prove their credibility with nothing more than a nodding acquaintance. In turn, the temporary nature of TPTs could increase abusive supervision climate perceptions due to less-developed trust with supervisors in such a short period of time. In addition, the findings showed that TPT membership flexibility enhances followers’ cognition-based trust in their ephemeral superiors. This trust is fostered through high role clarity established by a fine-grained standard procedure training and qualification-based role assignment. Successively, membership flexibility can decrease PASC by raising role-based trust. This study adds to the work of Goetz et al. [11], which proposed that employees who generate swift trust under pressure in temporary organizations would be more person-group compatible within temporary teams. Hence, the second contribution of this study was to shed light on the underlying mechanisms that link the temporary organizational features to subordinates’ sense of an abusive supervision climate through the mediating effect of trust in ad-hoc supervisors.

The third theoretical development of the study relates to the role of subordinate gender. Past research has widely viewed gender as a control variable when investigating supervisory abuse [62,72]. Several studies have demonstrated that abusers’ gender has a significant effect on abusive supervision incidence, as well as victims’ responses to abusive behaviors [53,73]. Our research revealed that subordinate gender is a significant moderator that influences the establishment of trust and the level of reported abusive supervision climate within a temporary work unit. This point is of the utmost importance as subordinates must make quick decisions about trust and promptly appraise supervisory misconduct as abusive or abrasive, given their involvement in transitory and fluctuating relationships. In such a stressful scenario, gender cues are the most salient indicators that subordinates may use to gauge their propensity to trust an unfamiliar supervisor.

The findings suggested that the negative connection between temporariness and trust in ad-hoc supervisors is only significant for female employees. As previously stated, trust can be categorized into cognitive trust and affective trust [46]. Cognitive trust is based on the functional attributes of the other party, such as competence, honesty, integrity, quality, and dependability. Affective trust develops as a result of a long-term social bond. McAllister [51] argued that cognition-based trust is crucial at the onset of a relationship, whereas affect-based trust becomes more salient as the relationship progresses. When a TPT grouping process is temporary, subordinates lack time to learn about each other’s interests, personality, or idiosyncrasies. The awareness of impending termination inhibits both parties from investing in a relationship. A short-term connection tends to be associated with less benefit reciprocation and a lower value of friendship. As a result, little affect-based trust can develop through TPTs’ transience compared to cognition- or competence-related trust.

In line with gender-role socialization theory [52], females associated with a communal or relationship-based pattern concentrate more on sustained exchanges and social intimacy [56]. Therefore, they place a higher premium on affective trust than cognitive trust. This suggests that the negative effect of temporariness on trust in ad-hoc supervisors is more pronounced for female subordinates.

Furthermore, the findings suggest that the positive relationship between membership flexibility and trust in ad-hoc supervisors was only validated for male workers. Men, in contrast to women, are frequently portrayed as agentic (i.e., instrumental or outcome-oriented) [54]. They tend to be more focused on the task at hand in task-oriented small groups, whereas females tend to be more social. Temporary work conditions generate an economic environment in which supervisor–subordinate interactions are more contractual than relational. Hence, males will place greater emphasis on the value of cognition-based trust, as it is more relevant to competence in achieving goals in this demanding setting. Membership resilience in TPTs achieved by a high level of role clarity (i.e., the extent to which employees understand their and others’ role expectations and responsibilities in the organization) [46] will help employees to understand subordinate and supervisory roles, which bolsters followers’ confidence in leaders’ work-related competence. As a result, a greater degree of membership flexibility increases cognitive trust, especially within ad-hoc contexts.

Lastly, our research discovered that the negative connection between trust and a perceived abusive supervision climate is more salient to female employees as opposed to their male counterparts. It is reasoned that males typically place a greater emphasis on extrinsic rewards, such as salary and benefits, whereas females are more motivated by social affirmation and workplace friendships [60]. As such, even if males have less trust in supervisors, they may prioritize autonomy over social interactions. Thus, males are more likely to view supervisory abusive behaviors as inconsequential in comparison to mission accomplishment within the time limit. In contrast, females with less affective trust in supervisors are more likely to identify supervisory abuse in economic exchanges. The study therefore advances social role theory by addressing what happens when genders interact in a transactional context.

### 5.2. Practical Implications

This research has significant implications for management practice in the airline industry and other analogous sectors that employ temporary grouping forms. First, it indicates that membership flexibility based on role clarity enhances subordinates’ cognitive trust in supervisors, leading to a lower perceived abusive supervision climate. Human resource practitioners should prioritize the implementation of institutionalized socialization strategies, specifically focusing on role-based group collaboration training. This approach enhances employee job dexterity and simultaneously reduces supervisor–subordinate role ambiguity. Using content strategies like sequential and fixed practices can inform staff of task sequences and promote confidence in supervisory competency, reducing perceptions of abusive climates. Moreover, new hires for TPTs should be selected based on shared values of autonomy and adaptability in order to maximize the person–organization fit for demanding temporary working scenarios.

Second, the study suggests that the temporal determination of TPTs makes it difficult to develop strong affective trust in ad-hoc supervisors. Therefore, human resource management professionals may need to promote affect-based trust by organizing get-togethers between supervisors and staff members [45]. By facilitating activities like company picnics, potlucks, and family outings, these programs may allow leaders and followers to interact informally and talk about topics outside of work. This can serve as a mediator and allow both parties to form emotional attachments.

Third, intervention strategies should be designed with subordinate gender in mind, as this research indicates that different genders value various aspects of trust and evaluate abusive supervisory behaviors differently. Therefore, when assigning members to a working group by roster systems, gender balance must also be considered in addition to qualifications and rest time. Counseling programs, assistance services, and extra organizational support should be provided to female subordinates in order to increase their self-reliance and reduce stress to work within alien and continually changing work relationships. Additional unofficial meetings may be helpful for female subordinates to construct their trust in ad-hoc superiors. These steps will assist staff, particularly female staff, to manage interpersonal stressors such as abusive supervision with greater confidence and efficacy.

### 5.3. Limitations and Further Research

The following limitations are discussed to pave a way for future research. First, due to topic sensitivity and difficulties in coordinating with airlines, ensuring random sampling and collecting sufficient data at Level 2 was challenging. Therefore, this study measured constructs at the individual level instead of the group level (e.g., shared perceptions within temporary professional teams) [63]. It is acknowledged that using individual perceptions to operationalize an abusive supervision climate may not fully capture the intrinsic characteristics and shared beliefs of an organization. Multi-level analyses are recommended to enhance research reliability. However, a one-way analysis of variance on airline ownership factors showed non-significant between-group variance for perceived abusive supervision climate (*p* > 0.05), reinforcing confidence in our findings and minimizing concerns about group-level effects.

Another limitation of our study is the reliance on self-reports from subordinates to gather data, which is consistent with previous research [41,63]. However, data were collected from multiple sources (flight attendants and pilots), and a multigroup comparison analysis yielded consistent results, reducing concerns about common method variance effects and increasing confidence in our findings. Additionally, we used carefully worded items in the PASC scale without directly mentioning ‘abuse’. This approach aimed to reduce deliberate misreporting and biases by alleviating respondents’ concerns about being associated with the socially undesirable group (the victim) [74].

Furthermore, the dataset used in this study was constrained to airline employees, which limits the generalizability of the findings beyond the aviation sector. The study only focused on the relationship between two temporary organizational characteristics and perceived abusive supervision climate, considering the temporary and flexible nature of aircrew configurations. Exploring other features of TPTs, such as member heterogeneity, is worth considering as it may influence subordinates’ perceptions of supervisory abuse. Surface-level diversity has been found to influence cohesiveness and social integration in temporary or ad-hoc groups [75]. Members would have relied on surface-level demographic data as an information proxy before deep-level ramification was achieved through long-term exchanges with their supervisors. Consequently, surface-level differences like ethnicity, race, nationalities, or native origins may be more relevant in studying the connection between this organizational feature and abusive supervision in temporary work settings. As the respondents for this study were Asian aircrews, there was limited variance in demographic characteristics. Cross-cultural studies examining the association between surface-level dissimilarity and abusive supervision among TPT members are warranted.

## 6. Conclusions

In the face of contemporary globalization, organizations are increasingly adopting agile and ambidextrous grouping forms. This study offers a novel viewpoint on the contextual factors influencing abusive supervision in temporary work units. We emphasize that not only sustained supervisory abuse but also intermittent misconduct can contribute to subordinates’ perceptions of an abusive supervision climate. Our research investigates the effects of two temporary organizational characteristics on perceived abusive supervision climate, with a focus on the mediating role of trust in ad-hoc supervisors. Additionally, this study explores gender interactions within these relationships.

Despite its limitations, our study contributes to the existing literature on abusive supervision and gender dynamics by examining an integrative moderated mediation model. It underscores that temporary structures and gender differences can influence employees’ evaluations of non-persistent abuse. We hope that our findings will inspire future scholars to consider the impact of intermittent supervisory abuse in flexible work environments and encourage further research to expand the theoretical framework by exploring other contextual antecedents and boundary conditions. Such endeavors will deepen our understanding of abusive supervision phenomena.

## Figures and Tables

**Table 1:** Means, standard deviations, and correlations among the research variables.

Variables	Mean	Std. Deviation	1	2	3	4	5	6	7	8
1. Age	2.96	0.51	1.00							
2. Job type	0.56	0.50	−0.09	1.00						
3. Tenure	4.15	2.57	0.59 **	0.20 **	1.00					
4. Subordinate gender	0.52	0.50	−0.05	0.92 **	0.15 **	1.00				
5. Temporariness	3.43	0.97	0.07	0.198 **	0.05	0.18 **	1.00			
6. Membership flexibility	4.19	0.66	0.10	−0.04	0.00	−0.05	0.29 **	1.00		
7. Trust in ad-hoc Supervisors	3.38	0.82	0.11	−0.39 **	−0.01	−0.38 **	−0.17 **	0.13 *	1.00	
8. Perceived abusive supervision climate	1.86	0.62	0.03	0.28 **	0.13*	0.30 **	0.27 **	−0.12 *	−0.53 **	1.00

Notes: ** Correlation is significant at the 0.01 level (two-tailed). * Correlation is significant at the 0.05 level (two-tailed).

**Table 2:** Measurement model results.

Model	χ2	df	∆χ2	CFI	TLI	RMSEA	SRMR
Hypothesized model: Alternative 4 (four-factor model) a	435.45	221	-	0.95	0.95	0.05	0.05
Alternative 3 (three-factor model) b	947.12	224	511.67 ***	0.84	0.82	0.10	0.08
Alternative 2 (two-factor model) c	1141.30	227	705.85 ***	0.80	0.78	0.11	0.10
Alternative 1 (one-factor model) d	1385.96	228	950.51 ***	0.75	0.72	0.12	0.11

Note. CFI = Comparative Fit Index, TLI = Tucker–Lewis Index, RMSEA = root-mean-square error of approximation. (a) Four-factors: Perceived abusive supervision climate; Temporariness; Membership flexibility; Trust in ad-hoc supervisors. (b) Three-factors: PASC; Temporariness; Membership flexibility and Trust in ad-hoc supervisors combined. (c) Two-factors: PASC; Temporariness, Membership flexibility and Trust in ad-hoc supervisors combined. (d) One-factor: PASC, Temporariness, Membership flexibility, Trust in ad-hoc supervisors all combined. *** *p* < 0.001 (two-tailed).

**Table 3:** The discriminatory validity analyses of potential variables.

Latent Variables	1	2	3	4
1. Perceived abusive supervision climate	***0.85***			
2. Trust in ad-hoc supervisors	−0.57	***0.78***		
3. Membership flexibility	−0.20	0.16	***0.65***	
4. Temporariness	0.32	−0.19	0.35	***0.73***

Notes: The square root of average variance extracted for each construct is denoted in bold and italic, while the inter-construct correlations are shown diagonally.

**Table 4:** The mediating effects results.

Path Relationships	Point Estimate	Product of Coefficient		Bootstrapping					
				Bias-Corrected 95% CI			Percentile 95% CI		
		SE	Z	Lower	Upper	*p*	Lower	Upper	*p*
Indirect Effects									
Hypothesis 2 (IE1): TEM → TRUSTS → PASC	0.09	0.03	3.15	0.04	0.15	***	0.04	0.15	***
Hypothesis 4 (IE2): MEMF → TRUSTS → PASC	−0.13	0.05	−2.77	−0.24	−0.05	***	−0.23	−0.05	**
Direct Effects									
DE1: TEM → PASC	0.19	0.05	4.31	0.12	0.29	***	0.11	0.29	***
DE2: MEMF → PASC	−0.25	0.10	−2.59	−0.45	−0.07	**	−0.45	−0.06	**
Total Effects									
TE1 = IE1 + DE1	0.28	0.05	5.17	0.19	0.40	***	0.18	0.39	***
TE2 = IE2 + DE2	−0.38	0.10	−3.87	−0.59	−0.21	***	−0.58	−0.20	***

Note: TEM, temporariness; MEMF, membership flexibility; TRUSTS, trust in ad-hoc supervisors; PASC, perceived abusive supervision climate; ** *p* < 0.01; *** *p* < 0.001.

**Table 5:** Hierarchical regression analyses.

	Dependent Variables									
	Trust in Ad–Hoc Supervisors				Perceived Abusive Supervision Climate					
	M1	M2	M3	M4	M1	M2	M3	M4	M5	M6
Step 1. Control variables										
Age	0.04	0.04	0.05	0.08	0.02	0.01	−0.01	−0.02	0.02	0.04
Job types	−0.40 ***	−0.36 ***	−0.22	−0.23	0.22 ***	0.16 **	−0.08	−0.08	−0.19	−0.13
Tenure	0.05	0.05	0.04	0.01	0.07	0.07	0.09	0.10	0.11	0.09
Step 2. Main effects										
Temporariness (TEM)		−0.15 **	−0.15 **	−0.01		0.29 ***	0.29 ***	0.26 **	0.25 ***	0.25 ***
Membership flexibility (MEMF)		0.16 **	0.15 **	0.37 **		−0.21 ***	−0.21 ***	−0.31 ***	−0.14	−0.21 **
Step 3. Main effect										
Subordinate gender (SG)			−0.16	−0.14			0.26	0.25	0.19	0.43 **
Step 4. Moderating effects										
TEM × SG				−0.16 *				0.04	−0.04	−0.06
MEMF × SG				−0.27 **				0.13	0.00	0.06
Step 5. Mediating effect										
Trust in ad-hoc supervisors (TRUSTS)									−0.46 ***	−0.27 ***
Step 6. Moderating effect										
TRUSTS × SG										−0.32 **
Overall F	21.03	15.54	13.21	13.16	7.45	11.75	10.51	8.30	18.10	17.60
R2	0.16	0.19	0.19	0.24	0.60	0.15	0.16	0.17	0.33	0.35
ΔR2	0.15	0.18	0.18	0.22	0.05	0.14	0.14	0.15	0.31	0.33

Note: M, Model. Cell values are standardized regression coefficients; * *p* < 0.05, ** *p* < 0.01, *** *p* < 0.001.

## Data Availability

Given the sensitivity of this topic, the data presented in this study are available on request from the corresponding author. The data are not publicly available due to privacy and ethical considerations.
